# Predicting illness progression for children with lower respiratory infections in primary care: a prospective cohort and observational study

**DOI:** 10.3399/BJGP.2022.0493

**Published:** 2023-11-14

**Authors:** Paul Little, Taeko Becque, Alastair D Hay, Nick A Francis, Beth Stuart, Gilly O’Reilly, Natalie Thompson, Kerenza Hood, Michael Moore, Theo Verheij

**Affiliations:** Primary Care Population Sciences and Medical Education Unit, University of Southampton, Southampton, UK.; Primary Care Population Sciences and Medical Education Unit, University of Southampton, Southampton, UK.; Centre for Academic Primary Care, Bristol Medical School: Population Health Sciences, University of Bristol, Bristol, UK.; Primary Care Population Sciences and Medical Education Unit, University of Southampton, Southampton, UK.; Primary Care Population Sciences and Medical Education Unit, University of Southampton, Southampton, UK.; Primary Care Population Sciences and Medical Education Unit, University of Southampton, Southampton, UK.; Primary Care Population Sciences and Medical Education Unit, University of Southampton, Southampton, UK.; Centre for Trials Research, College of Biomedical and Life Sciences, Cardiff University, Cardiff, UK.; Primary Care Population Sciences and Medical Education Unit, University of Southampton, Southampton, UK.; Julius Center for Health Sciences and Primary Care, University Medical Center Utrecht, Utrecht, the Netherlands.

**Keywords:** antibiotic resistance, antibiotics, respiratory tract infections, children, primary health care

## Abstract

**Background:**

Antibiotics are commonly prescribed for children with lower respiratory tract infections (LRTIs), fuelling antibiotic resistance, and there are few prognostic tools available to inform management.

**Aim:**

To externally validate an existing prognostic model (STARWAVe) to identify children at low risk of illness progression, and if model performance was limited to develop a new internally validated prognostic model.

**Design and setting:**

Prospective cohort study with a nested trial in a primary care setting.

**Method:**

Children aged 6 months to 12 years presenting with uncomplicated LRTI were included in the cohort. Children were randomised to receive amoxicillin 50 mg/kg per day for 7 days or placebo, or if not randomised they participated in a parallel observational study to maximise generalisability. Baseline clinical data were used to predict adverse outcome (illness progression requiring hospital assessment).

**Results:**

A total of 758 children participated (*n* = 432 trial, *n* = 326 observational). For predicting illness progression the STARWAVe prognostic model had moderate performance (area under the receiver operating characteristic [AUROC] 0.66, 95% confidence interval [CI] = 0.50 to 0.77), but a new, internally validated model (seven items: baseline severity; respiratory rate; duration of prior illness; oxygen saturation; sputum or a rattly chest; passing urine less often; and diarrhoea) had good discrimination (bootstrapped AUROC 0.83, 95% CI = 0.74 to 0.92) and calibration. A three-item model (respiratory rate; oxygen saturation; and sputum or a rattly chest) also performed well (AUROC 0.81, 95% CI = 0.70 to 0.91), as did a score (ranging from 19 to 102) derived from coefficients of the model (AUROC 0.78, 95% CI = 0.67 to 0.88): a score of <70 classified 89% (*n* = 600/674) of children having a low risk (<5%) of progression of illness.

**Conclusion:**

A simple three-item prognostic score could be useful as a tool to identify children with LRTI who are at low risk of an adverse outcome and to guide clinical management.

## INTRODUCTION

Lower respiratory tract infection (LRTI) is a frequent trigger for attendance in primary care, where nearly all children are managed, most receiving antibiotics.^1^^–^^4^ Antibiotics probably have limited benefit for LRTI in children,^5^ and individuals using antibiotics are subsequently likely to have more antibiotic- resistant organisms and prolonged infections.^6^^,^^7^ Outpatient antibiotic prescribing is strongly related to antimicrobial resistance (AMR).^8^ AMR is a global public health threat as much of modern medicine (for example, complicated infections, cancer care, and surgery) relies on antibiotics.^9^^,^^10^

Judicious and targeted use of antibiotics is likely to be key to both efficient clinical care and minimising the risk of antibiotic resistance. Clinicians are risk averse to a child developing a complication when a prescription has not been issued^11^ and so commonly prescribe antibiotics ‘in case’.^12^^–^^14^ A key problem is the absence of evidence to assist clinicians in the consultation to assess risk — to help tailor patient information, inform monitoring of disease, or help the targeting of treatment — particularly to minimise the use of antibiotics.^15^^–^^19^ The CRB-65 rule was developed in hospital settings to predict 30-day mortality, but is not well validated even among adults in primary care settings and is not suitable for use in children.^20^ There is one evidence- based, internally validated prognostic tool for children with LRTI developed in primary care — the STARWAVe model. This was developed in children presenting in primary care in the UK to predict hospital admissions for respiratory infection in the following 30 days post-consultation.^21^ The independently predictive variables included in the model were age <2 years, current asthma, illness duration of ≤3 days, parent- reported moderate or severe vomiting in the previous 24 h, parent-reported severe fever in the previous 24 h or a body temperature of ≥37.8°C at presentation, clinician-reported intercostal or subcostal recession, and clinician-reported wheeze on auscultation. The area under the receiver operating characteristic (AUROC) was 0.81 (the internally validated bootstrapped estimate). Although the model was internally validated, the spectrum of illness among children was milder than recent studies,^5^^,^^21^ and STARWAVe was not able to use oxygen saturation data, which are likely to be an important independent predictor of outcome.^16^^,^^17^

**Table:** How this fits in

Lower respiratory tract infection is one of the commonest presentation of children, and children commonly receive antibiotics, which fuels antibiotic resistance. Clinicians need to be able to identify children who are at low risk of illness progression but there are few prognostic tools available. In a cohort of unwell children presenting with uncomplicated LRTI an existing rule (the STARWAVe prognostic model) had limited performance in predicting illness progression. A new internally validated prognostic score (using respiratory rate, oxygen saturation, and having sputum or a rattly chest) performed well and could be useful in identifying children at low risk of adverse outcomes and guiding management.

Trial data alone can be less useful in generating prognostic models because of the inevitable selection bias (where more unwell children tend to be excluded), but nesting trial data within observational studies increases external validity of analyses and facilitates the generation of more robust clinical prediction models. The current study reports data on the progression of illness from a cohort of children that combines trial and observational data. Both the external validation of an existing prognostic model, and since the performance of the existing model was limited in this cohort of more unwell children, the development of a new internally validated model are reported.

## METHOD

### Study design

This was a cohort study including both children from a trial and a parallel observational study.

### Overview of methods

Full details of all data-collection methods have been previously published.^5^ Children were recruited between the ages of 6 months and 12 years presenting to primary care in UK general practices with acute uncomplicated LRTI, that is, where the clinician does not suspect pneumonia on clinical grounds. Parents and children were consented for participation by the responsible clinicians (usually GPs).

Acute LRTI was defined syndromically in several previous cohorts and trials as an acute cough as the predominant symptom, judged by the GP to be infective in origin, lasting <21 days (which will exclude children with protracted bacterial bronchitis^22^^,^^23^), and with other symptoms or signs localising to the lower respiratory tract (shortness of breath, sputum, and pain).^5^^,^^24^^–^^29^ Exclusion criteria included acute illness requiring immediate referral to hospital (for example, pneumonia and sepsis), non-infective causes of cough (for example, hay fever), immune deficiency, and inability to provide consent.

Where parents and clinicians were willing for children to be randomised, they were randomised to receive amoxicillin 50 mg/kg/day in divided doses for 7 days or placebo using pre-prepared packs randomised using a computer-generated random number by an independent statistician.^5^

Children not randomised (because they were ineligible or because of clinician or parent choice) were invited to participate in an observational study where the baseline clinical data and all outcome data as for the trial were collected by the same methods.^5^ In the observational study the choice of treatment was at the physician’s discretion and could involve antibiotic prescription or no prescription. Most practices that recruited children to the trial also recruited to the observational study but some sites could only recruit to the observational study.

### Outcomes

Progression of illness, the focus of this article, was defined as illness requiring hospital assessment and/or admission within 1 month of the index consultation. It was documented from a medical record review.^29^

### Sample size

The standard rules used by statisticians suggest that the number of variables that can be assessed robustly in a prognostic model is one variable per ten cases — so, with 29 children experiencing adverse outcomes the three-variable model should be adequately powered.^30^ However, the traditional rule of thumb has been questioned^30^ — and making the recommended assumptions^30^ (a margin of error of ≤0.05, a mean absolute prediction error of ≤0.05, and shrinkage factor of ≤10%, and expected optimism factor of 0.05), an expected outcome event rate of 5% suggested that 566 participants were required.

### Statistical analysis

Logistic regression models were used to assess the prediction of illness. The external validity of the STARWAVe score was assessed using the three STARWAVe classifications of low, normal, or high risk.^21^

As the performance of STARWAVe was limited in this population, the authors of the current study then developed a new model using a backwards fitting approach to model the predictors of progression of illness in the current dataset. Backward elimination is preferred among automatic predictor selection techniques because the correlations between predictors are accounted for in the multivariable model.^31^ Simulation studies suggest that *P*-values >0.05 should be considered, particularly in small datasets.^32^ For the first, most inclusive model the authors retained in the multivariable model the variables with *P*<0.20. Given the danger of overfitting, a model was also assessed for the variables that were significant at *P*<0.05 in multivariate analysis in the first model resulting in a four-item model, and finally a model was generated including the three most significant variables.

Developing a model with only a few major predictors can increase applicability in clinical practice. The authors therefore explored whether progression of illness could be predicted from the following baseline characteristics: age, the severity of symptoms, heart rate, respiratory rate, temperature, duration of illness before consultation, sex, comorbid conditions, history of asthma, chest signs, feeling generally unwell, oxygen saturation, sputum or a rattly chest, vomiting, dry cough, chills, diarrhoea, disturbed sleep, and passing urine less often.

The discrimination of the model in the AUROC is presented using bootstrapping to limit overly optimistic estimates because of overfitting, and a recommended and more efficient approach to internal validation rather than using split samples.^33^ Model calibration was assessed with a Hosmer– Lemeshow (HL) test.

## RESULTS

**Figure 1.**
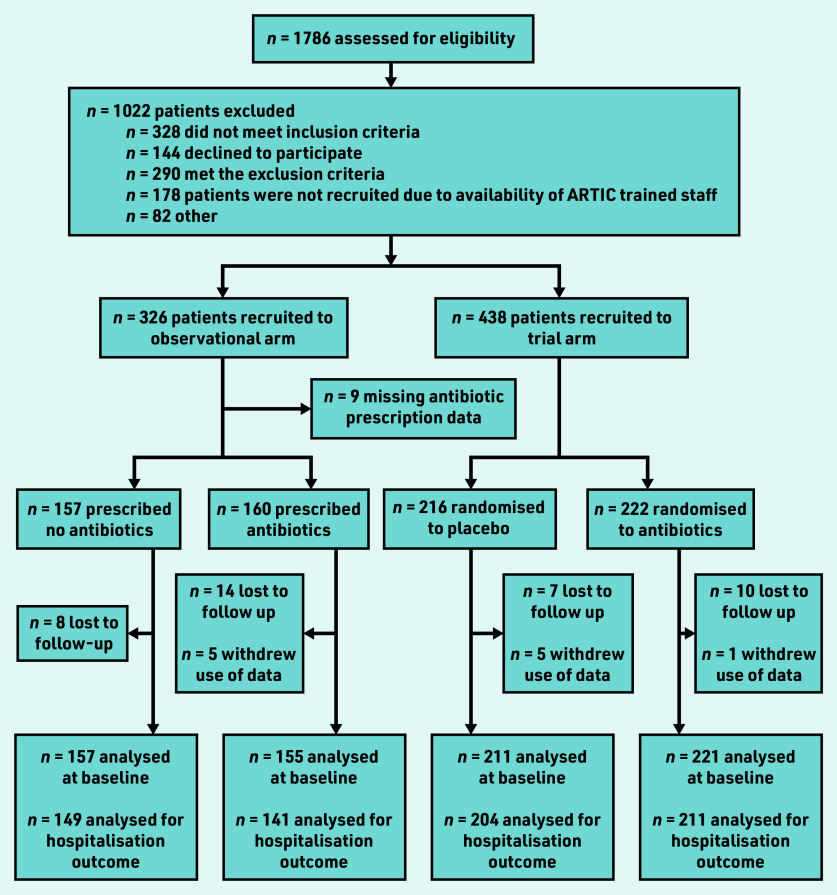
*Flow of patients through observational study and trial. ARTIC = Antibiotics for lower Respiratory Tract Infection in Children.*

A total of 326 patients were recruited to the observational study (Figure 1), 312 with antibiotic prescription data: 157 received no antibiotic, 141 immediate, and 14 delayed antibiotics. As the numbers with a delayed prescription were so small, these participants have been combined with the immediate prescription group for the purpose of analyses. Combined with the trial data, there were 744 participants in total, of whom 368 received no antibiotic and 376 were given or prescribed an antibiotic.

In the observational cohort, 52/312 (16.7%) were recruited via accident and emergency (A&E)/paediatric assessment versus 260/312 via GP practices. In the trial, 5/432 (1.2%) were recruited via A&E/paediatric assessment versus 427/432 via GP practices.

### Clinical characteristics

As expected, the number of children in the observational cohort with more severe clinical features (Table 1) was greater in the antibiotic group compared with the no antibiotic group — with more severe average baseline symptom scores (1.8 versus 1.5) for the antibiotic versus no antibiotic group, and more with abnormal chest signs (81.3% versus 24.2%), sputum or a rattly chest (87.1% versus 68.8%), fever during illness (91.0% versus 63.7%), feeling unwell (80.6% versus 51.0%), shortness of breath (70.3% versus 36.3%), and oxygen saturation <95% (21.2% versus 6.6%).

**Table 1.** Baseline characteristics of observational participants and combined dataset

**Characteristic**	**Observational study, *n* (%)^a^**		**Trial only, *n* (%)^a^**		**Combined, *n* (%)^a^**	

	**No antibiotics (*n* = 157)**	**Antibiotics (*n* = 155)**	**Placebo (*n* = 211)**	**Antibiotics (*n* = 221)**	**No antibiotics^b^ (*n* = 368)**	**Antibiotics (*n* = 376)**
**Male**	82 (52.2)	86 (55.5)	112 (53.1)	121 (54.8)	194 (52.7)	207 (55.1)

**Age, years (median, IQR)**	3.0 (1.4–4.9)	3.1 (1.8–5.2)	3.1 (1.4–5.6)	3.2 (1.7–5.8)	3.1 (1.4–5.4)	3.2 (1.7–5.5)

**Comorbidity^c^**	17 (10.8)	18 (11.6)	31 (14.7)	24 (10.9)	48 (13.0)	42 (11.2)

**Asthma**	9 (5.7)	10 (6.5)	19 (9.0)	13 (5.9)	28 (7.6)	23 (6.1)

**Long-term illness^c^**	12 (11.8)	7 (7.4)	7 (6.3)	13 (10.8)	19 (16.8)	20 (9.3)
Missing	55	61	100	101	255	162

**Baseline severity,^d^ mean (SD)**	1.5 (0.3)	1.8 (0.4)	1.6 (0.3)	1.6 (0.3)	1.6 (0.3)	1.7 (0.3)

**Duration of illness, days, median (IQR)**	5 (3–7)	4 (2–7)	6 (3–10)	5 (3–10)	6 (3–10)	5 (3–8)

**Abnormal chest signs^e^**	38 (24.2)	126 (81.3)	72 (34.1)	78 (35.3)	110 (29.9)	204 (54.3)

**Sputum or a rattly chest**	108 (68.8)	135 (87.1)	155 (73.8)	170 (77.6)	263 (71.7)	305 (81.1)
Missing	—	—	1	2	1	—

**Fever during illness**	100 (63.7)	141 (91.0)	161 (76.3)	177 (80.1)	261 (70.9)	318 (84.6)

**Unwell**	79 (51.0)	125 (80.6)	141 (66.8)	143 (64.7)	220 (60.1)	268 (71.3)
Missing	2	—	—	—	2	—

**Shortness of breath**	57 (36.3)	109 (70.3)	95 (45.0)	104 (47.1)	152 (41.3)	213 (56.6)

**Oxygen saturation <95%**	7 (6.6)	28 (21.2)	9 (5.4)	13 (7.6)	16 (5.9)	41 (13.6)
Missing	51	23	45	51	96	74

**STARWAVe^f^**						
Very low risk	94 (59.9)	60 (38.7)	110 (52.1)	123 (55.7)	204 (55.4)	183 (48.7)
Normal risk	60 (38.2)	77 (49.7)	95 (45.0)	94 (42.5)	155 (42.1)	171 (45.5)
High risk	3 (1.9)	18 (11.6)	6 (2.8)	4 (1.8)	9 (2.4)	22 (5.9)

**Physician rating of unwell,^g^ mean (SD)**	4.9 (1.9)	6.3 (1.6)	5.5 (1.7)	5.5 (1.6)	5.3 (1.8)	5.9 (1.7)

**Parent rating of unwell,^g^ mean (SD)**	3.3 (1.6)	5.3 (1.7)	3.8 (1.7)	3.7 (1.7)	3.6 (1.7)	4.3 (1.8)

**Temperature, mean (SD)**	37.1 (0.7)	37.5 (0.9)	37.3 (0.8)	37.2 (0.8)	37.2 (0.8)	37.3 (0.8)

**Oxygen saturation, mean (SD)**	97.6 (1.5)	96.1 (2.3)	97.3 (1.6)	97.3 (1.6)	97.4 (1.6)	96.8 (2.0)

**Heart rate, beats per min, mean (SD)**	110.8 (19.0)	124.5 (21.3)	112.0 (20.3)	111.8 (17.9)	111.6 (19.8)	117.1 (20.3)

**Respiratory rate, breaths per min, mean (SD)**	24.0 (7.4)	30.7 (10.3)	24.8 (6.8)	25.4 (7.1)	24.4 (7.0)	27.6 (8.9)

**Capillary refill >3 s**	1 (0.6)	3 (1.9)	3 (1.4)	2 (0.9)	4 (1.1)	5 (1.3)

**Consciousness**						
Normal	154 (98.7)	138 (90.2)	203 (96.2)	214 (97.3)	357 (97.3)	352 (94.4)
Irritable	1 (0.6)	11 (7.2)	8 (3.8)	5 (2.3)	9 (2.5)	16 (4.3)
Drowsy	1 (0.6)	4 (2.6)	0 (0.0)	1 (0.5)	1 (0.3)	5 (1.3)
Missing	1	2	—	1	1	3

**Ill appearance**	17 (10.8)	71 (45.8)	48 (22.7)	47 (21.3)	65 (17.7)	118 (31.4)

a *Unless otherwise stated.*

b *No antibiotics for the combined dataset comprises no antibiotics for the observational data and placebo for the trial data.*

c *Comorbidity includes asthma, heart disease, renal disease, diabetes, and other chronic disease. Longer-term illness was a self-report item in the diary to the question ‘Does he/she have any long-term illness, health problem, or illness/disease which limits his/her daily activities?’.*

d *Average baseline severity score for all symptoms: dry cough, wet cough, barking cough, wheezy cough, sputum or a rattly chest, runny/blocked nose, breathing faster than normal, wheeze/whistling chest, fever, chills/shivering, diarrhoea, vomiting, fewer fluids than usual, disturbed sleep, passing urine less often, headache, muscle aches, confusion, and sore throat (each on a scale 1 to 4: 1 = none, 2 = mild, 3 = moderate, and 4 = severe).*

e *Abnormal chest signs include wheeze, stridor, grunting, nasal flaring, inter/subcostal recession, crackles/crepitations, and bronchial breathing.*

f *STARWAVe prediction rule for hospital admission (short illness, temperature, age, recession, wheeze, asthma, and vomiting).*

g *Physician and parent rating of unwell on a scale 0 to 10. IQR = interquartile range. SD = standard deviation.*

Progression of illness was recorded for 705 of 744 participants (94.8%). A total of 29 (4.1%) children in the cohort had illness progression requiring attendance or admission to hospital (for details of the illnesses, see Supplementary Box S1).

### Prognostic model

The STARWAVe score was tested using the three classifications. The calibration was excellent with a HL test *P*-value of 0.9847, but the AUROC was modest 0.66 (95% confidence interval [CI] = 0.50 to 0.77). As STARWAVe was developed to predict progression of illness requiring overnight hospital admission (11 of the children in the current dataset), test performance for this outcome was separately tested for. The AUROC for STARWAVe in predicting the need for overnight hospital admission was 0.70 (95% CI = 0.56 to 0.84; HL *P* = >0.99) (data not shown).

Given the modest discrimination of the STARWAVe model in this population, the authors of the current study developed a new model. The predictors of the progression of illness are shown in Table 2. The variables that were retained in the model were baseline severity; difference in respiratory rate from normal for age; oxygen saturation <95%; sputum or a rattly chest; passing urine less often or drier nappies than normal; diarrhoea; and duration of illness before consultation. The model had an AUROC of 0.83 (95% CI = 0.74 to 0.92) and model calibration was good with a HL *P*-value of 0.55 (data not shown). A four- item model including significant variables (at the 5% level) from the seven- item model (difference in respiratory rate from normal for age; presence of sputum or a rattly chest; oxygen saturation <95%; and passing urine less frequently or drier nappies than normal) had an AUROC of 0.83 (95% CI = 0.73 to 0.92) for progression of illness, with a HL *P*-value of 0.51.

**Table 2.** Predictor of progression of illness (requiring hospital attendance or admission) using the combined trial and observational datasets

**Characteristic**	**No progression of illness, *n* (%)^a^**	**Progression of illness, *n* (%)^a^**	**Univariable RR (95% CI)**	**Multivariable RR (95% CI)**
**Female**	316/676 (46.7)	10/29 (34.5)	0.57 (0.27 to 1.24)	—

**Age, mean (SD)**	3.79 (4.89)	3.59 (2.75)	1.00 (0.93 to 1.06)	—

**Baseline severity, mean (SD)**	1.64 (0.33)	1.73 (0.38)	2.29 (0.96 to 6.13)	0.44 (0.13 to 1.53)

**Longer duration of illness, days before consultation**	419/676 (62.0)	13/29 (44.8)	0.57 (0.27 to 1.17)	0.53 (0.23 to 1.23)

**≥1 comorbid condition**	83/676 (12.3)	4/29 (13.8)	1.03 (0.35 to 3.00)	—

**Asthma**	66/676 (9.8)	3/29 (10.3)	0.98 (0.29 to 3.30)	—

**Abnormal chest signs**	271/676 (40.1)	20/29 (69.0)	3.72 (1.67 to 8.29)	—

**Sputum or a rattly chest**	404/670 (60.3)	23/28 (82.1)	2.62 (1.05 to 6.50)	4.04 (1.45 to 11.27)

**Unwell**	442/676 (65.4)	24/29 (82.8)	1.89 (0.77 to 4.61)	—

**Oxygen saturation <95%**	43/509 (8.4)	10/28 (35.7)	5.87 (2.64 to 13.09)	2.39 (0.98 to 5.82)

**Dry cough**	368/676 (54.4)	12/29 (41.4)	0.64 (0.31 to 1.31)	—

**Runny nose**	550/676 (81.4)	24/29 (82.8)	1.17 (0.44 to 3.11)	—

**Diarrhoea**	89/676 (13.2)	5/29 (17.2)	1.28 (0.48 to 3.42)	1.85 (0.68 to 5.01)

**Chills**	165/676 (24.4)	7/29 (24.1)	1.02 (0.44 to 2.40)	—

**Vomiting**	217/676 (32.1)	14/29 (48.3)	2.22 (1.07 to 4.61)	—

**Taking less fluid**	284/676 (42.0)	16/29 (55.2)	1.72 (0.83 to 3.56)	—

**Disturbed sleep**	575/676 (85.1)	27/29 (93.1)	2.10 (0.50 to 8.71)	—

**Passing urine less often or drier nappies than normal**	165/676 (24.4)	15/29 (51.7)	3.71 (1.79 to 7.72)	2.60 (1.04 to 6.48)

**Temperature, mean (SD)**	37.26 (0.78)	37.5 (1.08)	1.35 (0.90 to 2.03)	—

**Heart rate, beats per min, mean (SD)**	113.5 (19.69)	125.8 (22.96)	1.03 (1.01 to 1.05)	—

**Difference between respiratory rate and normal respiratory rate for age, breaths per min, mean (SD)^b^**	−1.7 (7.8)	5.7 (11.5)	1.08 (1.05 to 1.11)	1.06 (1.02 to 1.10)

**Antibiotics**	331/676 (49.0)	20/29 (69.0)	2.64 (1.20 to 5.81)	N/A

**STARWAVe**				
Very low risk	363/676 (53.7)	8/29 (27.6)	Reference	N/A
Normal risk	292/676 (43.2)	17/29 (58.6)	2.70 (1.16 to 6.28)	—
High risk	26/676 (3.8)	4/29 (13.8)	7.60 (2.34 to 24.59)	—

a *Unless otherwise stated.*

b *‘Normal’ respiratory rate for age (breath per minute) taken as the midpoint from Fleming* et al*^34^ — 12 to 18 months: 35; 18 to 24 months: 31; 2 to 3 years: 28; 3 to 4 years: 25; 4 to 6 years: 23; 6 to 8 years: 21; 8 to 12 years: 19. N/A = not applicable. RR = risk ratio. SD = standard deviation.*

A reduced model using only the three significant predictors from the four-item model (difference in respiratory rate from normal for age; presence of sputum or a rattly chest; and oxygen saturation <95%; Table 3) had an AUROC of 0.81 (95% CI = 0.70 to 0.91) and HL *P*-value of 0.42 (data not shown). Antibiotic prescription was not included in the primary predictive model because of the observed inverse association between antibiotic prescribing and the progression of illness (very likely because of confounding by indication), nevertheless the model was also assessed after including antibiotic prescription and the same variables were still included. In addition, the discrimination of the four- item model for the 11 children requiring overnight admissions was looked at: the AUROC was 0.89 (95% CI = 0.80 to 0.97; HL *P* = 0.88). The AUCROC for the three-item model was 0.85 (95% CI = 0.72 to 0.99), but an HL *P*-value of 0.009 indicates a poor model fit for overnight admission.

**Table 3.** Predictors of progression of illness using the combined trial and observational datasets with reduced models

**Characteristic**	**No progression of illness, *n* (%)^a^**	**Progression of illness, *n* (%)^a^**	**Four-item model, RR (95% CI)**	**Three-item model, RR (95% CI)**
Difference between respiratory rate and normal respiratory rate for age, breaths per min, mean (SD)	−1.7 (7.8)	5.7 (11.5)	1.06 (1.02 to 1.10)	1.07 (1.03 to 1.11)
Sputum or a rattly chest	404/670 (60.3)	23/28 (82.1)	3.69 (1.34 to 10.10)	3.00 (1.29 to 7.01)
Oxygen saturation <95%	43/509 (8.4)	10/28 (35.7)	2.46 (1.03 to 5.87)	4.38 (1.62 to 11.84)
Passing urine less often	165/676 (24.4)	15/29 (51.7)	2.13 (0.89 to 5.07)	—
Diarrhoea	89/676 (13.2)	5/29 (17.2)	—	—
Baseline severity,^a^ mean (SD)	1.64 (0.33)	1.73 (0.38)	—	—
Longer duration of illness, days before consultation	419/676 (62.0)	13/29 (44.8)	—	—

a *Unless otherwise stated. RR = risk ratio. SD = standard deviation.*

The three-item model (difference in respiratory rate from normal for age; oxygen saturation <95%; and presence of sputum or a rattly chest) was converted to a score by multiplying the beta coefficients by 10 and rounding to nearest integer (score = 46 + difference in respiratory rate from normal for age + 14*low oxygen saturation + 18*sputum, where low oxygen saturation = 1 if oxygen saturation <95% [0 otherwise], and sputum = 1 for presence of sputum or rattly chest [0 otherwise]). Scores ranged from 19 to 101, and the AUROC was 0.78 (95% CI = 0.67 to 0.88) for progression of illness and 0.86 (95% CI = 0.72 to 1.00) for hospital admission (data not shown).

The performance of the score for different cut-off points is shown in Tables 4 and 5, and it is clear that the risk of progression of illness remains low (<5%) until a score of 70, as does the risk of overnight admission.

**Table 4.** Progression of illness and overnight admission for different cut-offs for the three-item prognostic score

**Score**	**No progression of illness, *n* (%), *N* = 646**	**Progression of illness, *n* (%), *N* = 28**	**Risk of progression, %**	**No overnight admission, *n* (%), *N* = 663**	**Overnight admission, *n* (%), *N* = 11**	**Risk of admission, %**
<40	49 (7.6)	0 (0.0)	0.0	49 (7.4)	0 (0.0)	0.0
40–49	177 (27.4)	4 (14.3)	2.2	180 (27.1)	1 (9.1)	0.6
50–59	161 (24.9)	2 (7.1)	1.2	163 (24.6)	0 (0.0)	0.0
60–69	199 (30.8)	8 (28.6)	3.9	205 (30.9)	2 (18.2)	1.0
70–79	43 (6.7)	3 (10.7)	6.5	44 (6.6)	2 (18.2)	4.4
80–89	12 (1.9)	7 (25.0)	36.8	14 (2.1)	4 (36.4)	26.3
≥90	5 (0.8)	4 (14.3)	44.4	8 (1.2)	2 (18.2)	11.1

**Table 5.** Sensitivity and specificity for different cut-offs of the three-item prognostic score

**Score cut-off**	**Progression of illness, %**				**Overnight admission, %**			
	**Sensitivity**	**Specificity**	**PPV**	**NPV**	**Sensitivity**	**Specificity**	**PPV**	**NPV**
≥40	100	7.6	4.5	100	100	7.4	1.8	100
≥50	85.7	35.0	5.4	98.3	90.9	34.5	2.3	99.6
≥60	78.6	59.9	7.8	98.5	90.9	59.1	3.6	99.7
≥70	50.0	90.7	18.9	97.7	72.7	90.0	10.8	99.5
≥80	39.3	97.4	39.3	97.4	54.5	96.7	21.4	99.2
≥90	14.3	99.2	44.4	96.4	9.1	98.8	11.1	98.5

*NPV = negative predictive value. PPV = positive predictive value.*

## DISCUSSION

### Summary

A simple three-item prognostic score using clinical variables that are readily available in the consultation could be useful as a tool to identify children with acute LRTI who are at low risk of significant illness progression to guide clinical management.

### Strengths and limitations

The differences in clinical characteristics between the observational and the trial data participants was expected, but cannot be attributed just to clinical decision making in deciding who to recruit to the trial or the observational study as some observational patients came from less typical sites that were not able to recruit to the trial (for example, A&E departments). The prognostic model was limited by the relatively few children where illness progressed (potentially limiting detection of relevant variables because of lower power), but using both traditional rules of thumb and more recent guidance the study should have had sufficient power, at least for the three-item model.^30^

The discrimination of the model was good, but the current study may have underestimated the discriminatory ability, as more significant variables would increase the discriminatory power. Bootstrapping limited the problem of overfitting and provided internal validation, which is the recommended most-efficient approach to internal validation rather than using split samples.^33^ The reduced model with fewer variables also provided reasonable estimates of discrimination, but external validation will be needed. The presence of clinician-diagnosed asthma was not a predictor of outcome, and the number of children with the diagnostic label of asthma was very few (<10%).

### Comparison with existing literature

Children given antibiotics in the observational study had much more severe clinical presentations than children not given antibiotics — matching the trends in STARWAVe.^21^^,^^35^ The children in both the trial and observational cohorts were more severely affected than STARWAVe — among children given antibiotics, very high percentages had sputum production (87% in the observational cohort compared with 63% in STARWAVe), fever (91% and 75%, respectively), and shortness of breath (70% and 46%, respectively).

Some evidence of external validation is provided in that STARWAVe is able to distinguish different levels of prognostic risk in this dataset, albeit with lower levels of prognostic accuracy than in the derivation dataset,^21^ which could be explained by the different populations studied. The new prognostic model to predict the progression of illness included seven variables and demonstrated good discrimination (AUROC 0.83) and calibration. However, it included rather different variables to the STARWAVe model,^21^^,^^35^ only age and prior illness duration being in common (STARWAVe included age <2 years; current asthma; illness duration ≤3 days; parent- reported moderate or severe vomiting in the previous 24 h; parent-reported severe fever in the previous 24 h or a body temperature ≥37.8°C at presentation; clinician-reported intercostal or subcostal recession; and clinician-reported wheeze on auscultation). Thus, it is perhaps not surprising that the STARWAVe model had lower discrimination in the current population (AUROC 0.66), and the differences probably reflect the more severely affected group of children recruited for the current study. Other differences with STARWAVe are: pulse oximetry was only available in <50% of children for the STARWAVe cohort, and if saturation had been more available it might have been included in STARWAVe; and intercostal recession was also not measured in the current study, so it is possible the estimates of discrimination might be improved further. If the outcome was ‘illness progression sufficient to require overnight hospital admission’, then the discrimination of both the new model and STARWAVe would have improved further.

### Implications for practice

A simple, internally validated clinical score for use in more unwell children with uncomplicated acute LRTI uses variables that are easily documented in routine consultations, and shows promise in enabling clinicians to identify the minority of children whose illness is more likely to progress sufficiently to require hospital assessment, and those where the illness is very unlikely to progress. The clinical score has the potential to help guide management decisions to minimise antibiotic use for children at low risk and/or for closer follow up for children at high risk.^17^ The clinical score preferably also requires external validation and only applies to children with acute LRTI; children with protracted bacterial bronchitis (a wet cough lasting >4 weeks, commonly recurrent) are likely to benefit from a longer course of antibiotics.^22^^,^^23^ The simplicity of the rule, using variables that do not require extensive medical training, means that there is potential for the use of the clinical score in settings such as pharmacies — but this would require careful assessment of feasibility and safety. Although the data come from a UK setting, the clinical score is also likely to be valid for children presenting with LRTI in other high-income countries, but may not be valid in low- and middle- income countries, even when oxygen saturation monitors are available.

## Data

De-identified participant data are available for further analyses. Request for data, with justification, should be made to the corresponding author.
